# Using a knowledge exchange event to assess study participants’ attitudes to research in a rapidly evolving research context

**DOI:** 10.12688/wellcomeopenres.15651.2

**Published:** 2020-08-28

**Authors:** Iona Beange, Elizabeth J. Kirkham, Sue Fletcher-Watson, Matthew H. Iveson, Stephen M. Lawrie, G. David Batty, James P. Boardman, Ian J. Deary, Corri Black, David J. Porteous, Andrew M. McIntosh

**Affiliations:** 1 Division of Psychiatry, Centre for Clinical Brain Sciences, University of Edinburgh, Edinburgh, EH10 5HF, UK; 2 Department of Epidemiology and Public Health, University College London Medical School, London, WC1E 7HB, UK; 3 School of Biological and Population Health Sciences, Oregon State University, Corvallis, Oregon, USA; 4 Medical Research Council Centre for Reproductive Health, University of Edinburgh, Edinburgh, EH16 4TJ, UK; 5 The Lothian Birth Cohorts, Division of Psychology, University of Edinburgh, Edinburgh, EH8 9JZ, UK; 6 Institute of Applied Health Sciences, University of Aberdeen, Aberdeen, AB25 2ZD, UK; 7 MRC Institute of Genetics and Molecular Medicine, University of Edinburgh, Edinburgh, EH4 2XU, UK

**Keywords:** Public Engagement, Cohort, Data Science, Data Linkage, Opinion, Guthrie, Health, Scotland, Knowledge Exchange, Big Data

## Abstract

**Background: **The UK hosts some of the world’s longest-running longitudinal cohort studies, which make repeated observations of their participants and use these data to explore health outcomes. An alternative method for data collection is record linkage; the linking together of electronic health and administrative records. Applied nationally, this could provide unrivalled opportunities to follow a large number of people in perpetuity. However, public attitudes to the use of data in research are currently unclear. Here we report on an event where we collected attitudes towards recent opportunities and controversies within health data science.

**Methods: **The event was attended by ~250 individuals (cohort members and their guests), who had been invited through the offices of their participating cohort studies. There were a series of presentations describing key research results and the audience participated in 15 multiple-choice questions using interactive voting pads.

**Results: **Our participants showed a high level of trust in researchers (87% scoring them 4/5 or 5/5) and doctors (81%); but less trust in commercial companies (35%). They supported the idea of researchers using information from both neonatal blood spots (Guthrie spots) (97% yes) and from electronic health records (95% yes). Our respondents were willing to wear devices like a ’Fit-bit’ (88% agreed) or take a brain scan that might predict later mental illness (73%). However, they were less willing to take a new drug for research purposes (45%). They were keen to encourage others to take part in research; whether that be offering the opportunity to pregnant mothers (97% agreed) or extending invitations to their own children and grandchildren (98%).

**Conclusions: **Our participants were broadly supportive of research access to data, albeit less supportive when commercial interests were involved. Public engagement events that facilitate two-way interactions can influence and support future research and public engagement efforts.

## Introduction

The UK has hosted some of the world’s longest running longitudinal cohort studies of health and wellbeing. These long-term projects make repeated observations of their participants and use these data to explore how factors such as health, wealth, family, and education influence health outcomes and mortality. Together, these studies have led to several thousand publications (e.g. Generation Scotland, 2019; Lothian Birth Cohort, 2019; UK Biobank, 2019), and to policy changes that have impacted national and global health ( Pearson, 2017; Power & Elliott, 2006).

Cohort studies are, nevertheless, highly resource intensive and subject to participant attrition. It is also difficult to make them future-proof; for example, it is not possible to anticipate every measure that may become of interest to researchers in the future. Furthermore, inevitable changes to lifestyle and technology can make cohort data collected decades ago less relevant to current circumstances.

An alternative method to longitudinal ‘face-to-face’ follow-up of individual participants is record linkage: the linking together of electronic health and administrative records, which are routinely collected (e.g. as part of a hospital visit or census). Although not collected with research in mind, these records can nevertheless be combined to produce a comprehensive and longitudinal dataset. If applied nationally, this type of dataset could provide unrivalled opportunities for researchers to follow a large number of people in perpetuity. Data linkage also has several advantages over face-to-face follow up, not least the fact that it is participant-passive and of negligible burden to the participant. This in turn reduces study attrition and increases the representativeness of study findings. Record linkage is also more flexible than face-to-face assessments, as it can be updated to capture new events, exposures and outcomes.

A recent extension to record linkage studies, particularly in Denmark and Sweden, has been the identification and analysis of dried neonatal blood spots. These were originally obtained as heel-prick neonatal blood samples and used to detect inborn errors of metabolism. Nick-named ‘Guthrie Spots’ after the physician who devised them, these dried blood spots have been collected and archived by NHS Scotland since 1965; and now number around 3 million in total. In Denmark, there is a long-established biobank of newborn blood spots that is available for anonymised research ( Norgaard-Pedersen & Hougaard, 2007). However, such research access has not yet been granted in Scotland. Nevertheless, Generation Scotland have demonstrated the feasibility of using adults dried blood spots for DNA methylation studies, and have shown that they can accurately replicate the findings made with fresh peripheral blood ( Walker *et al*., 2019). Thus, the NHS Scotland blood spot archive has a high potential research value.

Nevertheless, record linkage studies, including those that use archived blood spots, also have several drawbacks. These include their dependence upon administrative recording processes, which may not be standardised within large organisations like the NHS. It is important to consider that administrative records are not collected with research in-mind, and data may be of lower quality or need substantial pre-processing before it can be used. Furthermore, the systems and legal basis for the use of archived data and samples may vary depending on which organisation is responsible for their retention. Even in situations where the data and samples are available for research, it would be impossible to obtain informed consent from all of the individuals to whom the data and samples relate.

Public attitudes to the use of such data and samples for research are currently unclear. It is not known what proportion of the public are aware of their retention, their value and whether they would approve of their diversion for approved forms of research. It is also unclear whether the public would approve the use of samples such as blood spots for all research, and if so with what sort of regulatory oversight and approval mechanism?

Similarly, researchers are interested in public opinion on other tricky issues such as: Should children be allowed to consent to their own participation in research? Who would you trust with your data? Should predictive brain scans be offered for later mental illness?

Each of the individual questions above could be investigated using in-depth processes such as a citizen jury. However, these processes are both time-consuming and expensive. We were therefore interested to see if any useable data could be gathered using a simpler voting pad procedure. Furthermore, we were particularly interested in the views our specific cohort members, whose data we analyse and whom we have the capacity to invite to take part in additional research studies. Here we report on an event at which we sought to engage with individuals and their families from across diverse Scottish research cohorts. We aimed to both share our recent research findings and to assess attitudes towards recent opportunities and controversies on topics such as: electronic health record linkage; the repurposing of biological samples for research use; and the involvement of commercial interests (amongst other topics). By collecting these opinions from our cohort members, we sought to better understand their views and to provide a basis for further public engagement on these issues. In particular, by asking individuals who had taken part in research to bring along a guest, we also sought to test whether the individuals who had participated in research differed in their attitudes towards data linkage and analysis when compared to those who had not.

Members of four cohorts were invited to the event:

**The Lothian Birth Cohort 1936**
  Members received their first intelligence test in 1947 as part of The Scottish Mental Survey which tested every 11-year-old in Scotland. They were then retraced in the early 2000’s. They have been taking part in face-to-face testing every 3 years since then. The team also make use of record linkage.
**The Aberdeen Children of the 1950s**
  Members took part in the Aberdeen Child Development Survey in 1962 and were retraced in late 1990s. Their data comes from record linkage and a postal survey sent out between 2001 and 2003.
**Generation Scotland**
  Participants were recruited from 2006 onwards and attended one appointment where blood, other samples, clinical measurements and information about their health and lifestyle were gathered. Most of the follow on data comes from record linkage although some participants have also been invited to take part in additional studies.
**Theirworld Edinburgh**
  Launched in 2015, they hope to monitor premature babies from birth to adulthood. They see the babies at birth and then again after nine months, two years, five years and then hope to see them every five years until they are 25.

## Methods

### Participants

This study reports on the purpose and findings for an ‘all cohorts’ meeting under the banner “A Celebration of Scottish Health Research: Participatory Research in Cohort Studies of Mental and Physical Health” held in Edinburgh on 10th June, 2018.

The event format and venue was based upon the successful ‘reunion’ model developed by Professor Ian Deary and his team at the, who regularly update their Lothian Birth Cohorts 1921 and 1936 members about their study findings.

The event was held at The Assembly Hall, Mound Place, Edinburgh. This is the meeting place of the General Assembly of the Church of Scotland and was previously home to The Scottish Parliament between 1999 and 2004. This meant that the venue had experienced technicians who could provide and install high quality presentation equipment, filming equipment and up to 600 interactive voting pads, allowing the collection of participant responses in real time.

Participants from a number of Scottish cohort studies (Aberdeen Children of the 1950s ( Leon *et al*., 2006), Generation Scotland ( Smith *et al*., 2013), Lothian Birth Cohort ( Deary *et al*., 2012), and Theirworld Edinburgh Birth Cohort ( TEBC, 2016)) were invited to attend an event at which they would hear key results from the studies in which they had participated. The event was also used as an opportunity to measure attitudes towards future research, including routine health record/sample linkage and its subsequent analysis.

Participants were personally invited using paper invitations which were posted out via their cohort managers or, in the case of Generation Scotland, via The Health Informatics Centre at The University of Dundee. (Data protection and GDPR laws meant it was not possible for us to obtain cohort members' names and addresses, so invitations could not be posted out directly.) The invitation is available as *Extended data* ( Beange *et al*., 2019). Selection for invitation was done by the cohort managers, based upon factors such as permission to re-contact, postcode, etc. All participants of the Lothian Birth Cohort were invited; for practical reasons, a randomly selected subsection of the other cohorts were invited.

### Event logistics

The event was attended by approximately 250 individuals. Upon arrival, participants received a delegate pack (a Centre for Cognitive Ageing and Cognitive Epidemiology branded cloth bag) which contained (amongst other things):

- A programme for the afternoon
- A filming and photography notice
- A Keep-in-touch form - to allow us to contact them again after the event
- A feedback form - to evaluate the event
- A list of stands
- Centre for Cognitive Ageing and Cognitive Epidemiology (CCACE) notes: Celebrating Participatory Research Magazine, with stories from each of the presenters (Available as Extended data, Beange *et al*., 2019).
- A SHARE Leaflet (Volunteer to share NHS records for research purposes) https://www.registerforshare.org/
- A trolley coin, pencil, pen and mints

**Figure 1.**
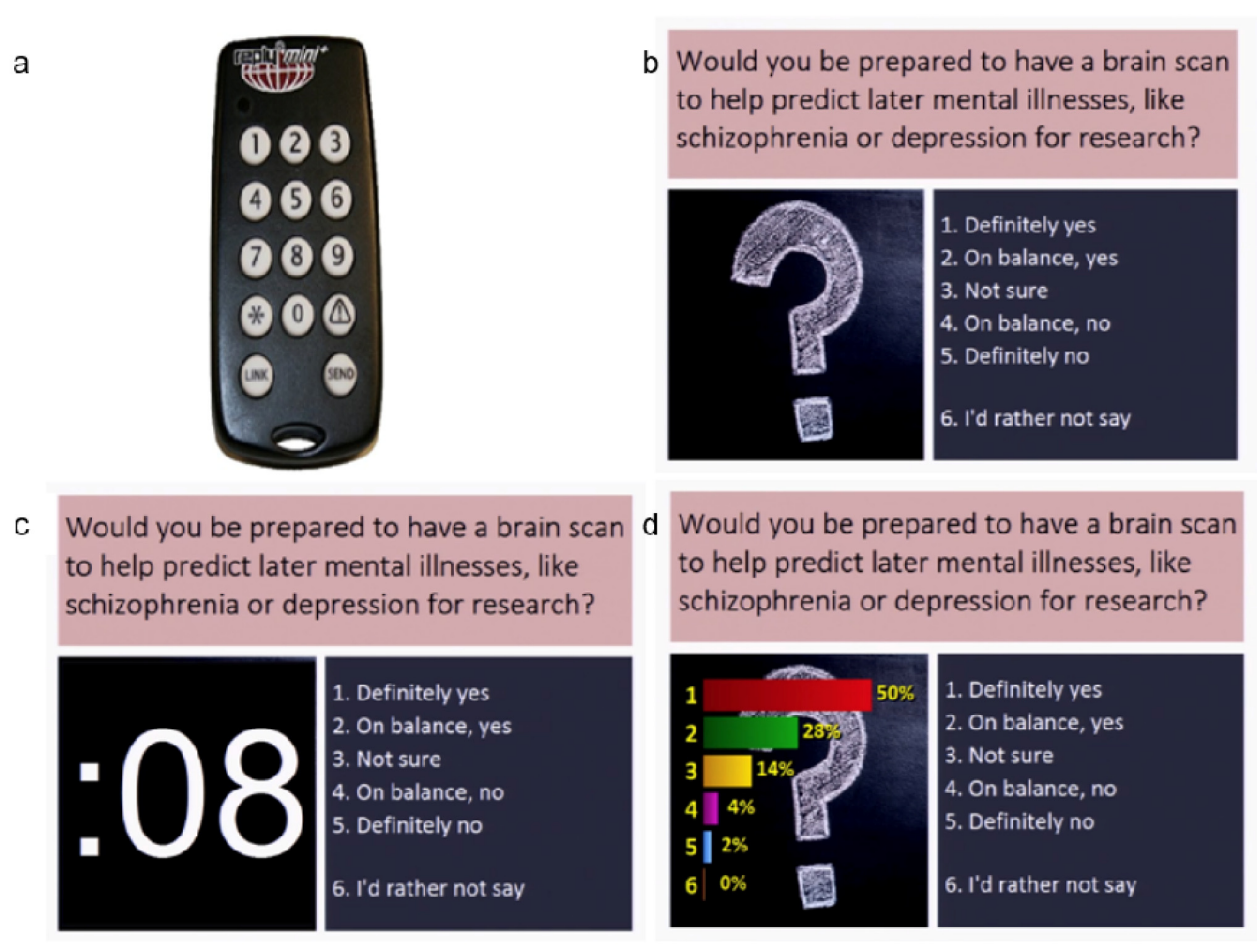
Voting system used. ( **a**) A photo of the interactive voting pad that was used. Other panels show the appearance of the screen at various time points during the voting procedure: ( **b**) when the question was asked; ( **c**) during voting, when a 10 second countdown appeared on screen; ( **d**) the result of the vote.

They also received an interactive voting pad on a lanyard (see Figure 1).

The number of voting attendees from each study, and their accompanying guests, are shown in Table 1. The majority of voting participants were cohort members, but 31% were guests (e.g. the partner, child or friend of a cohort member). *NB: Some participants who attended are not included in Table 1, either because they arrived late or because they did not use the voting pads.*

**Table 1.** Breakdown of cohort membership at the cohort event. These data were collected via the interactive voting pads. Not all attendees chose to participate in every question. Additionally, a few individuals arrived late or left early and did not provide complete data for every question.

Cohort	No. of attendees*	% attendees
Aberdeen Children of the 1950s & Generation Scotland	17	8%
Generation Scotland only	24	11%
Lothian Birth Cohort	99	47%
Theirworld Edinburgh Birth Cohort	4	2%
Guest	66	31%
I’d rather not say	1	1%
**Total**	**211**	

No other demographic information was collected from participants on the day, although we know that Lothian Birth Cohort members were approximately 82 years old and Aberdeen Children of the 1950s members were between 62 and 68 years old. Participants came from across all regions of Scotland.

Ethical permission was granted by The Psychology Research Ethics Committee (PREC) at the University of Edinburgh (Ref No: 327-1718/3). As no identifying data were collected from participating individuals, it was deemed that written consent to participate was not necessary. The information sheet given to participants is included in *Extended data* ( Beange *et al*., 2019). Participants had the option to take part (press clicker button) or not for every question as it arose.

Written photography and filming consent was obtained from all speakers, so the talks could be filmed and uploaded to the ccacevideo YouTube channel. (The videos and slides can also be found in the *Extended data*, Beange *et al*., 2019).

For the public, photography notices were displayed prominently on the walls and on seats which had the potential to be captured by photography or video recording. A more detailed photography notice, which indicated potential uses for the photographs/video was also included in the delegate pack. To comply with data protection regulations (GDPR), these notices included contact details to allow people to withdraw their consent after the event, should they wish to do so. Alternative seating was available for those who preferred not to be captured in this way.

### Event programme

The meeting began with a short introductory talk by Prof Andrew Morris, Vice Principal of Data Science at The University of Edinburgh, who outlined the importance of medical research and extended his thanks to the cohort members.

He was followed by Professor Andrew McIntosh, Professor of Biological Psychiatry at The University of Edinburgh, who introduced the concept of a health cohort study, set out how healthcare data was used in research and instructed the audience in the use of the voting system.

These introductory talks were then followed by 6 topic-specific presentations, each of approximately 20 minutes in duration (see Table 2), and which included 2-3 voting pad questions (see Table 3).

**Table 2.** List of talks. Each talk represents a different cohort. The talks can be viewed on the ccacevideo YouTube channel. The slides and videos can also be found in the Extended data files, Beange *et al*., 2019.

Presenter (order)	Presentation title	Brief description
**Prof J Boardman,** University of Edinburgh	**Growing up following** **premature birth**	**Theirworld Edinburgh Birth Cohort.** **Purpose**: to investigate the causes and consequences of being born too soon or too small on brain development and long term outcomes on children and their families.
**Professor Corri** **Black,** University of Aberdeen	**Whatever happened to the** **Aberdeen Children of the** **1950s?**	**The Aberdeen Children of the 1950s** Purpose: to study the determinants of health and ill health in a group of individuals born in Aberdeen in the 1950s
**Professor David** **Porteous,** University of Edinburgh	**Generation Scotland** **- Next Generation**	**Generation Scotland** **Purpose**: to conduct a family and population based study of genetic and environmental determinants of physical and mental health.
**Professor Stephen** **Lawrie,** University of Edinburgh	**Youth Mental Health in** **Families at High Risk**	**The Edinburgh High Risk Study and Bipolar Family Study** **Purpose**: to follow a group of unaffected young people at high genetic risk of schizophrenia or bipolar disorder and identify the baseline predictors and trajectories of those who would later become unwell.
**Professor Ian** **Deary,** University of Edinburgh	**Ten Lothian Birth Cohort** **Commandments**	**The Lothian Birth Cohort** The Lothian Birth Cohort study aims to examine non-pathological cognitive ageing and its determinants. Individuals born in 1921 and 1936 and living in the Lothians were first invited to participate in 1999. The cognitive ability and health of participants has been monitored as they have aged.
**Professor David** **Batty,** University College London	**Living Longer in Scotland**	**Combining Scottish and English Cohort Studies** For the last 4 decades the people of Scotland have experienced markedly shorter life expectancy than their English counterparts. We report on our attempts to understand the reasons for these differentials.

**Table 3.** List of multiple choice questions and voting responses. Each speaker asked 2 or 3 questions during or at the end of their talk. The questions are itemized in the order that they were asked and the potential multiple-choice answers for each question are listed. Explanations of technical terms were given with the question, or in the accompanying presentation.

Presentation Topic	Question	Response Options	Frequencies
Introduction	Which cohort do you belong to?	1. Aberdeen Children of the 50s and Gen Scotland	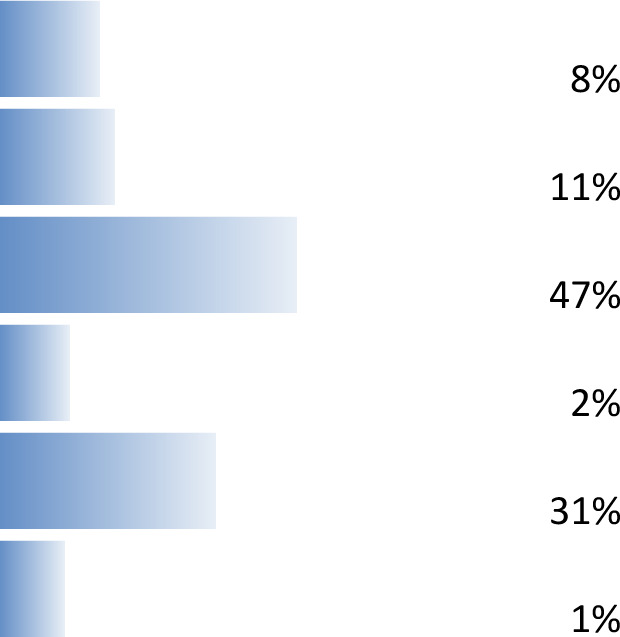
		2. Generation Scotland only	
		3. Lothian Birth Cohort	
		4. Theirworld Edinburgh Birth Cohort	
		5. I’m here as a guest/ I am not a member of a cohort	
		6. I’d rather not say (1 person)	
			[211 respondents]
Theirworld Edinburgh Birth	At what age do you think the issue of the child consenting to continued participation in a birth cohort study should be raised?	1. 10 years	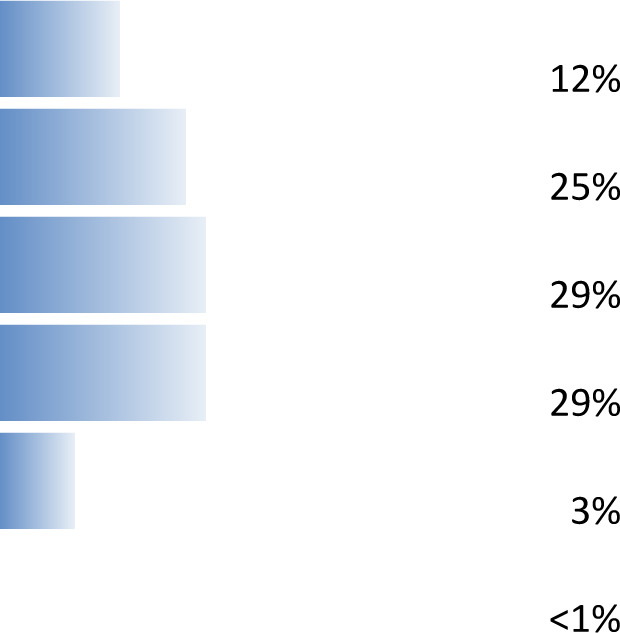
Cohort		2. 12 years	
(Paediatric cohort)		3. 14 years	
		4. 16 years	
		5. Not sure	
		6. I’d rather not say (1 person)	
			[201 respondents]
	Do you think that all pregnant women and their babies who receive care in the NHS should be offered an opportunity to contribute to knowledge and evidence by participating in approved research studies?	1. Definitely yes	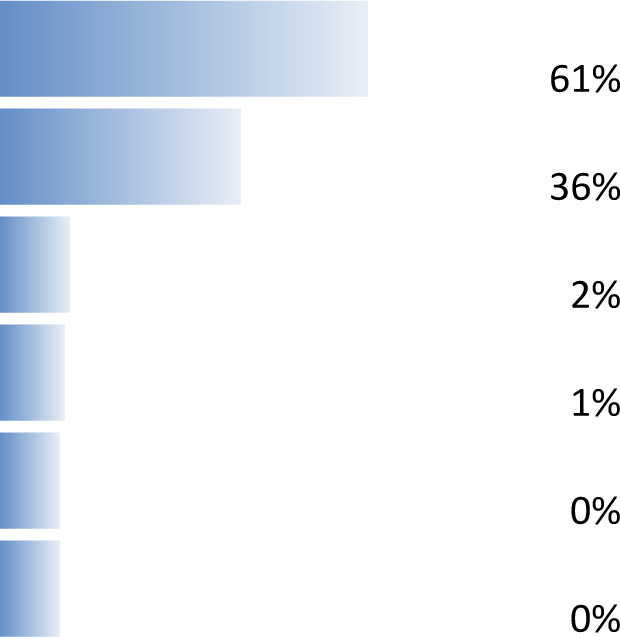
		2. On balance, yes	
		3. Not sure	
		4. On balance, no	
		5. Definitely no	
		6. I’d rather not say	
			[215 respondents]
	Do you think that approved researchers should be allowed access to these blood spots?	1. Definitely yes	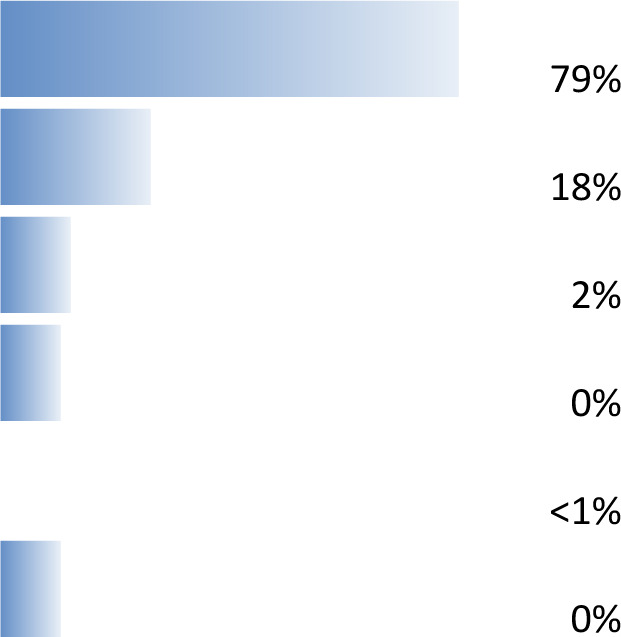
		2. On balance, yes	
		3. Not sure	
		4. On balance, no	
		5. Definitely no (1 person)	
		6. I’d rather not say	
			[216 respondents]
Aberdeen children of the 1950s	Would you be willing for researchers to use information from your health record in research?	1. Yes, without reservation	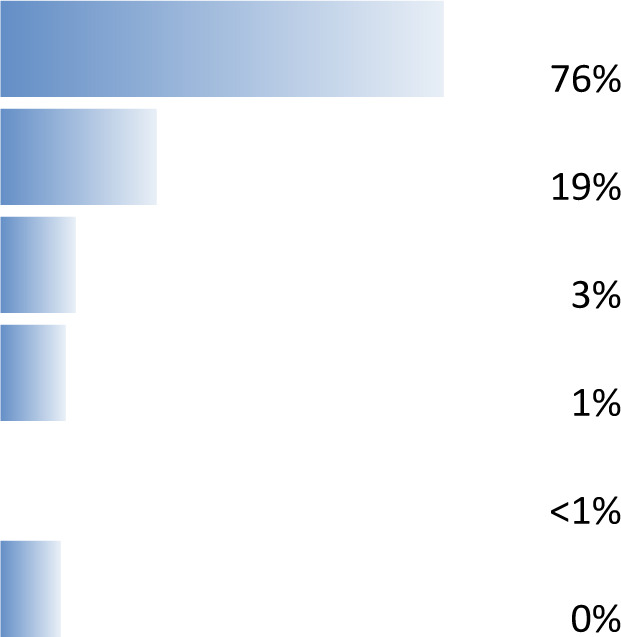
(Older age cohort)		2. On balance yes	
		3. Not sure	
		4. On balance, no	
		5. Without reservation no (1 person)	
		6. I’d rather not say – no votes	
			[214 respondents]
	We would like to collect information about how much and where you exercise using something like a watch or ‘Fit Bit’. Would you be willing?	1. Yes, without reservation	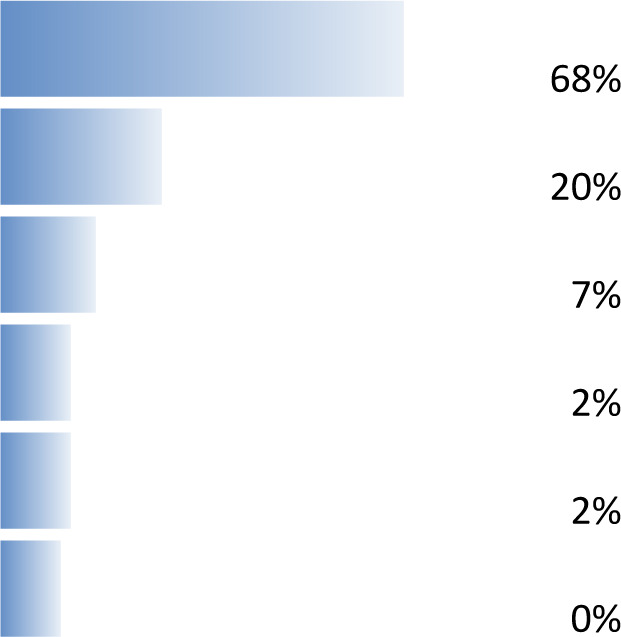
		2. On balance yes	
		3. Not sure	
		4. On balance, no	
		5. Without reservation no	
		6. I’d rather not say	
			[213 respondents]
Generation Scotland	On a scale of 1 (not very) to 5 (totally) how much do you trust University Health Researchers with your data?	1. (not very)	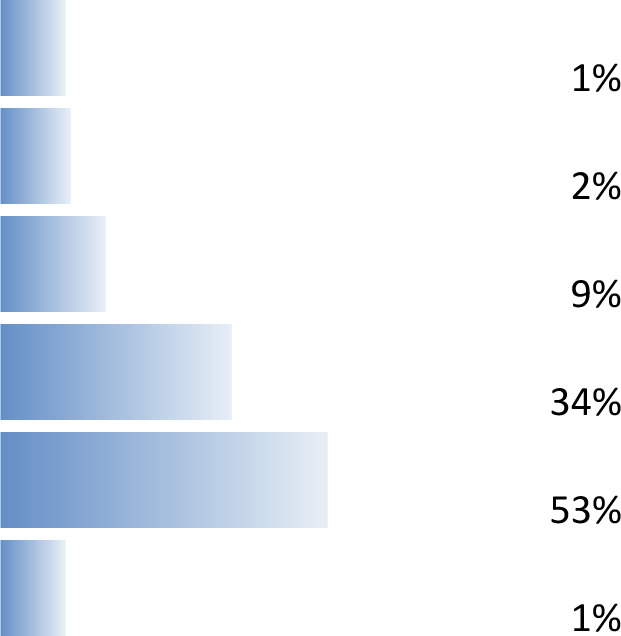
(Family cohort)		2.	
		3.	
		4.	
		5. (totally)	
		6. I’d rather not say (2 people)	
			[220 respondents]
	On a scale of 1 (not very) to 5 (totally) how much do you trust your GP or hospital doctor with your data?	1. (not very)	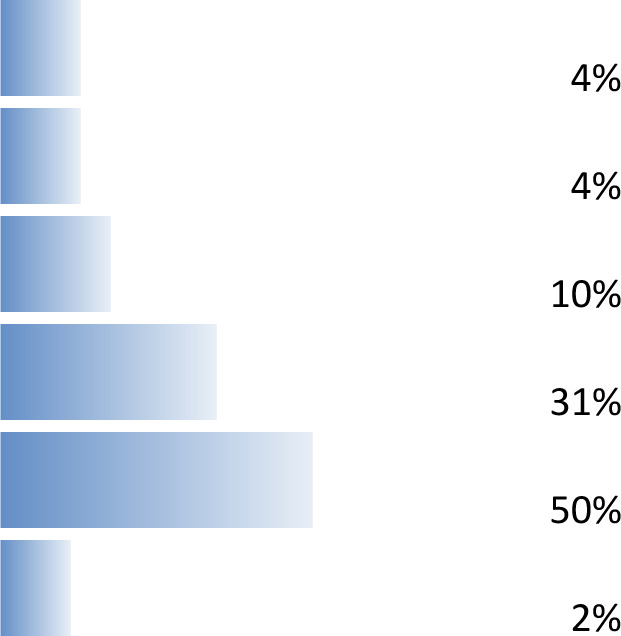
		2.	
		3.	
		4.	
		5. (totally)	
		6. I’d rather not say (4 people)	
			[220 respondents]
	On a scale of 1 (not very) to 5 (totally) how much do you trust companies developing new tests or drugs with your data?	1. (not very)	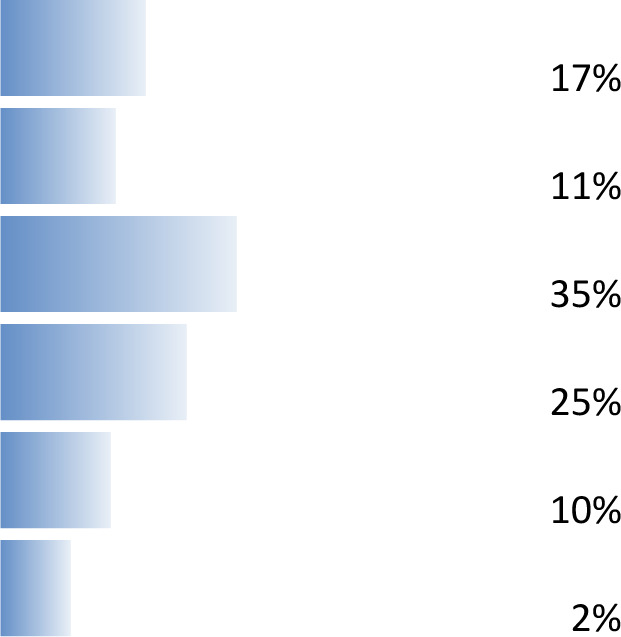
		2.	
		3.	
		4.	
		5. (totally)	
		6. I’d rather not say (4 people)	
			[213 respondents]
Youth Mental Health	Would you be prepared to have a brain scan to help predict later mental illnesses, like schizophrenia or depression for research?	1. Definitely yes	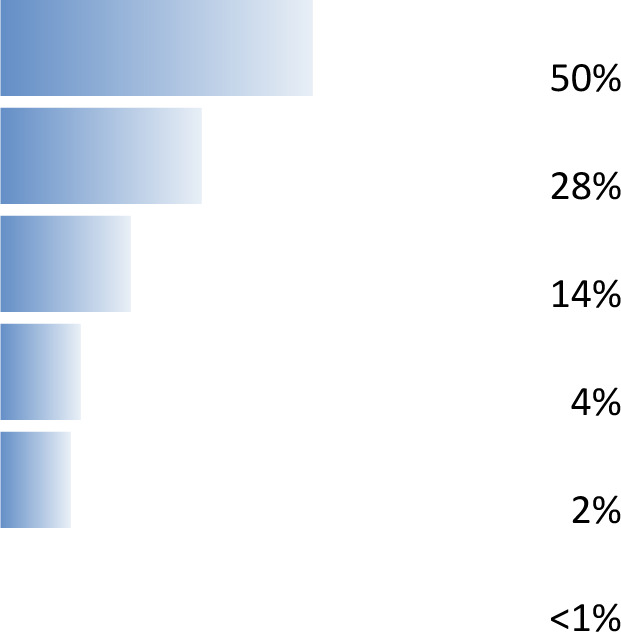
		2. On balance, yes	
		3. Not sure	
		4. On balance, no	
		5. Definitely no	
		6. I’d rather not say (1 person)	
			[202 respondents]
	Would you want to have access to a brain scan test of future mental illness, if it were safe and accurate?	1. Definitely yes	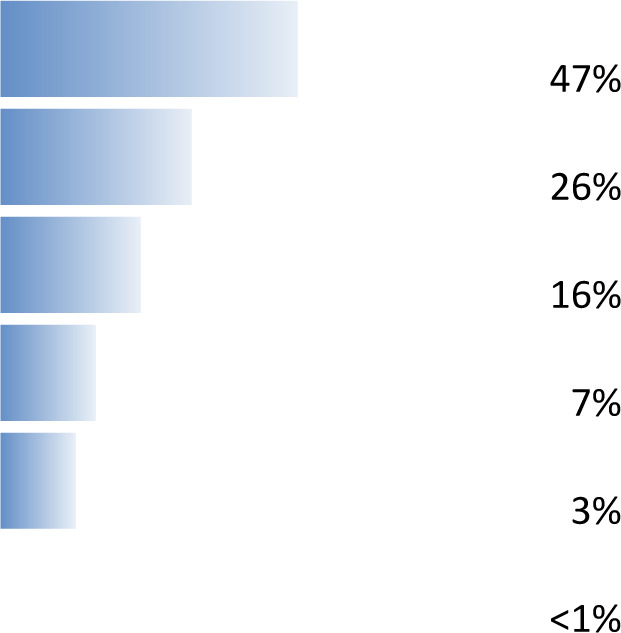
		2. On balance, yes	
		3. Not sure	
		4. On balance, no	
		5. Definitely no	
		6. I’d rather not say (1 person)	
			[205 respondents]
Lothian Birth Cohort	If asked, would you encourage your children and grandchildren to take part in research cohorts?	1. Definitely yes	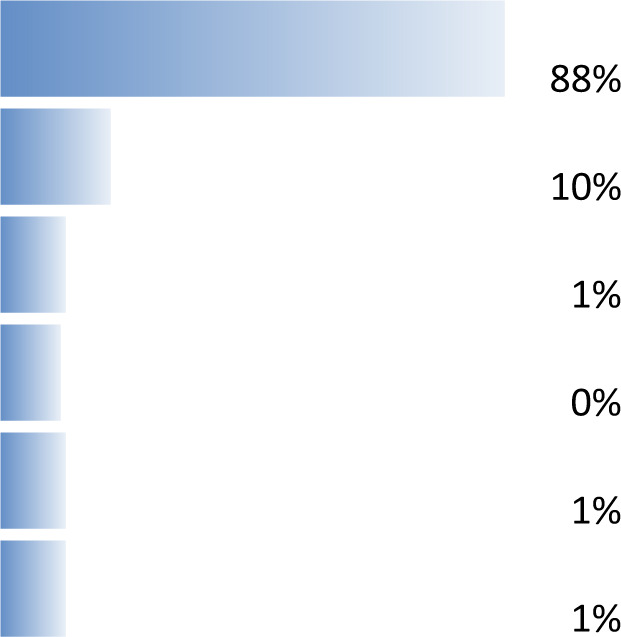
(Older age cohort)		2. On balance, yes	
		3. Not sure – 1% (1 person)	
		4. On balance, no – no votes	
		5. Definitely no (1 person)	
		6. I’d rather not say (1 person)	
			[199 respondents]
	If someone has said no, or not given a reply, [to post- mortem brain donation] should researchers approach them again to see if they have changed their mind/ would like to donate now?	1. Definitely yes, ask them again	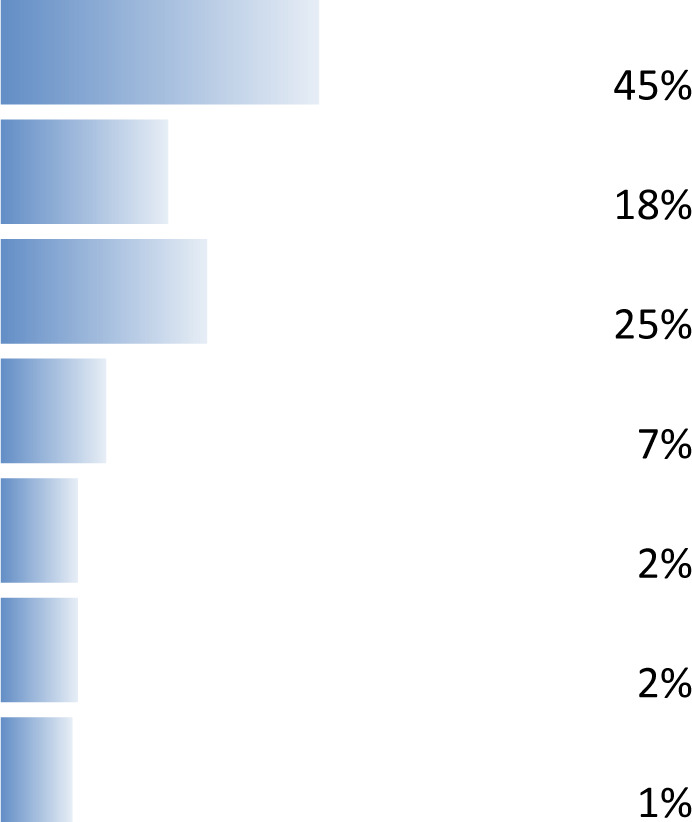
		2. Possibly yes	
		3. Yes, but only if they didn’t reply previously	
		4. Not sure	
		5. Probably not	
		6. Definitely not	
		7. I’d rather not say (2 people)	
			[189 respondents]
Combining Scottish and English cohort data.	Would you be willing to repeat the testing you have already done but on a more frequent basis? (i.e. every 2 years?)	1. Definitely yes	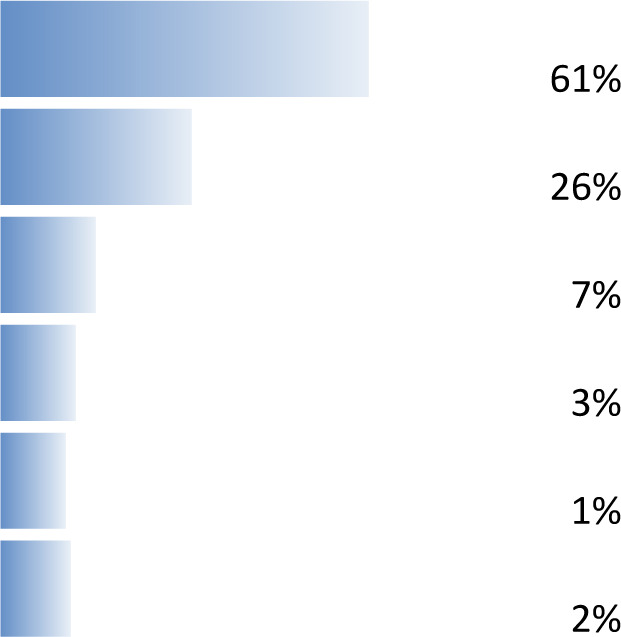
		2. On balance, yes	
		3. Not sure	
		4. On balance, no	
		5. Definitely no (2 people)	
		6. I’d rather not say (3 people)	
			[176 respondents]
	Would you be willing to change an aspect of your lifestyle (e.g. attend a social club, change your diet) as part of an intervention study?	1. Definitely yes	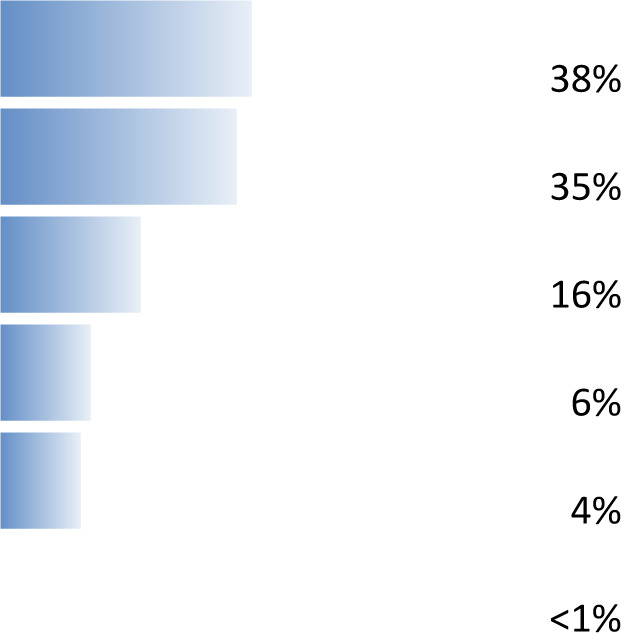
		2. On balance, yes	
		3. Not sure	
		4. On balance, no	
		5. Definitely no	
		6. I’d rather not say (1 person)	
			[188 respondents]
	Would you be willing to take a new drug as part of an intervention study?	1. Definitely yes	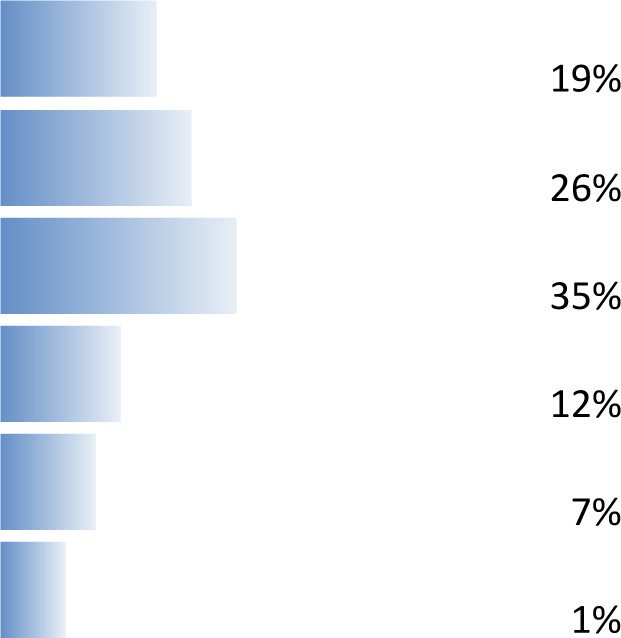
		2. On balance, yes	
		3. Not sure	
		4. On balance, no	
		5. Definitely no	
		6. I’d rather not say (1 person)	
			[186 respondents]

### Voting procedure

Multiple choice questions were posed by each speaker, during or at the end of their talk (see Table 3. Participants were given a list of the questions in their delegate packs (see also cohort meeting slide deck, *Extended data* ( Beange *et al*., 2019)). Participants had 10 seconds to respond via an interactive voting pad ( Figure 1). If they pressed more than one button, only their last result was recorded. When the vote closed, the results were immediately displayed on the screens ( Figure 1b–d). Participants could choose not to vote at any point. Videos of all talks given, as well as each of the slides used during these talks, are available as *Extended data* ( Beange *et al*., 2019).

### Statistical analysis

The majority of the results are reported as a percentage of total respondents. Where people answered both question 1 (cohort membership/guest) and one of the other questions, Mann-Whitney U-tests were used to compare groups (see Table 4).

**Table 4.** Statistical differences between guests and cohort participants. Table showing statistical comparisons. Mann-Whitney U-tests were used to compare the opinions of cohort members and guests.Results have not been adjusted for multiple comparisons. There is no indication that failure to answer one question had any impact on the next, so each question has been analysed individually. Cohort groups have been compared using Kruskal Wallis tests. Where the results were significant post-hoc pairwise comparisons were performed using Dunn's test and the Bonferroni correction for multiple tests applied. Significant results are highlighted in yellow and labelled. The number of people who answered each question is also listed. There are many reasons why people may not have answered a particular question including practical factors like arriving late or leaving early, or failing to press a button within the allocated 10 seconds. In addition, some people have been excluded from this analysis, because they did not answer question 1, therefore we cannot allocate them to the categories of ‘cohort member’ or ‘guest’.

				Comparing Cohort members to guests			Comparing Cohort Groups			
Question	No. Cohort Members	No. of Guests	Total (n)	Mann- Whitney U	Significance (p)	Notes	Kruskal Wallis (H)	Degrees of freedom	Significance (p)	Post-hoc pairwise comparisons (Dunn's test adjusted for multiple comparisons)
1.Which cohort/guest										
2. Age of consent	129	60	189	3346	0.121		7.322	3	0.062	
3. Pregnant Women	135	64	199	4111	0.518		12.246	3	0.007	ACONF more positive than LBC (p=0.020)
4. Blood spots	135	64	199	4102	0.413		11.206	3	0.011	ACONF more positive than Gen Scot (p=0.047)
5. Access health record	134	63	197	3738	0.081		4.987	3	0.173	
6. Fitbit	133	62	195	3888	0.427		4.697	3	0.195	
7. Trust researchers	133	64	197	3349	0.008	Cohort members more trusting than guests	4.328	3	0.228	
8. Trust doctors	135	63	198	3110	0.001	Cohort members more trusting than guests	8.823	3	0.032	No statistically significant results
9. Trust companies	134	61	195	3499	0.095		4.049	3	0.256	
10. Brain scan for research	128	56	184	3279	0.319		3.366	2	186	
11. Brain scan for health info	128	57	185	3256	0.209		1.224	2	0.542	
12. Invite children/ grandchildren	125	56	181	3460	0.825		2.322	2	0.313	
13. Reapproach re: post- mortem brain donation	121	51	172	2291	0.005	Cohort members more positive than guests	0.172	2	0.917	
14. Repeat testing more regularly	121	40	161	1886	0.015	Cohort members more positive than guests.	5.239	2	0.073	
15. Change lifestyle	125	46	171	2530	0.205		10.647	2	0.005	ACONF more positive than LBC (p=0.007)
16. Take new drug	122	47	169	2757	0.687		1.429	2	0.489	

Results were not adjusted for multiple comparisons. There is no indication that a failure to answer one question had any impact on the next, so each question has been analysed individually. There are many different reasons why people may not have answered a particular question including practical factors like arriving late or leaving early, or failing to press a button within the allocated 10 seconds.

In addition, some people have been excluded from this analysis, because they did not answer question 1, therefore we cannot allocate them to the categories of ‘cohort member’ or ‘guest’.

## Results

In total, 234 people voted at least once during the event and the number of responses to each question ranged from 176 to 220. Data are presented above in terms of frequency counts, and we examined in each case the difference in opinion between cohort participants and other event guests. Summary frequencies for the participants’ responses are shown in Table 3, organised according to the topic of the presentation that immediately preceded the questions being asked. Raw and summary results are available as *Underlying data* ( Beange *et al*., 2019).

### Summary of results

Overall, our respondents were very positive about health data research (See Table 3).

When asked if all pregnant women should be given the opportunity to take part in research, 97% of our respondents replied ‘yes’. But the response was more mixed when they were asked at what age a child participating in such a birth cohort should consent to continued participation, with a fairly even spread of results across 12, 14 and 16 years of age (25%, 29% and 29% respectively). A total of 12% of respondents suggested the age of consent should be as low as 10 years old.

Similarly, our respondents were very positive about researchers accessing data held by the NHS such as neonatal ‘Guthrie Spots’ (97% yes) and routinely collected health care records (95% yes).

On the issue of trust, our participants showed a high degree of trust in university health researchers (87% of participants scored them 4/5 or 5/5) and doctors (81% scored them 4/5 or 5/5). However, less trust was expressed for companies with commercial interests (only 35% scored them 4/5 or 5/5).

For research data collection purposes 88% of our respondents were willing to wear a ‘Fit-bit’ style activity monitor and 73% were willing to change an aspect of their lifestyle (e.g. attend a social club or change their diet). However, only 45% were willing to take a new drug as part of an intervention study. Nevertheless, 88% of our respondents were prepared to undergo a brain scan to help researchers predict later mental illness and 73% would like access to such a test more generally, if it were safe and accurate.

Post-mortem brain donation is an option for members of the Lothian Birth Cohort (LBC) and would be a valuable addition to the thinking skills tests and MRI scans that they currently take every 3 years. All members have already received a letter inviting them to donate their brain after death. However, positive responses have been low. In this question, we asked if LBC members should be approached again about this decision. Of our respondents, 45% said that cohort members should be approached again and a further 18% said ‘possibly yes’. We also offered a more nuanced option of ‘yes, if they didn’t reply before’ which 25% of our respondents selected. Of our respondents, 7% said they were ‘not sure’ and 4% said cohort members should not be contacted again about this option.

Encouragingly, 98% of our participants would encourage their children and grandchildren to take part in a research cohort.

Finally, we asked if participants would be willing to repeat the testing that they have already done, but on a more frequent basis (i.e. every 2 years). In total, 87% of respondents said yes, highlighting again our respondents’ high level of enthusiasm for health data research.

### Differences between cohorts

Cohort groups have been compared using Kruskal Wallis tests. Where the results were significant post-hoc pairwise comparisons were performed using Dunn's test and the Bonferroni correction for multiple tests applied. There was some indication that Aberdeen Children of the 1950’s had a more positive attitude to some of the questions, however, overall the groups were very similar in their responses (see Table 4).

### Differences between guests and cohort participants

Significant differences between cohort participants and their invited guests are described below. All test results (including non-significant results) are reported in Table 4. Results have not been adjusted for multiple comparisons.

**Figure 2.**
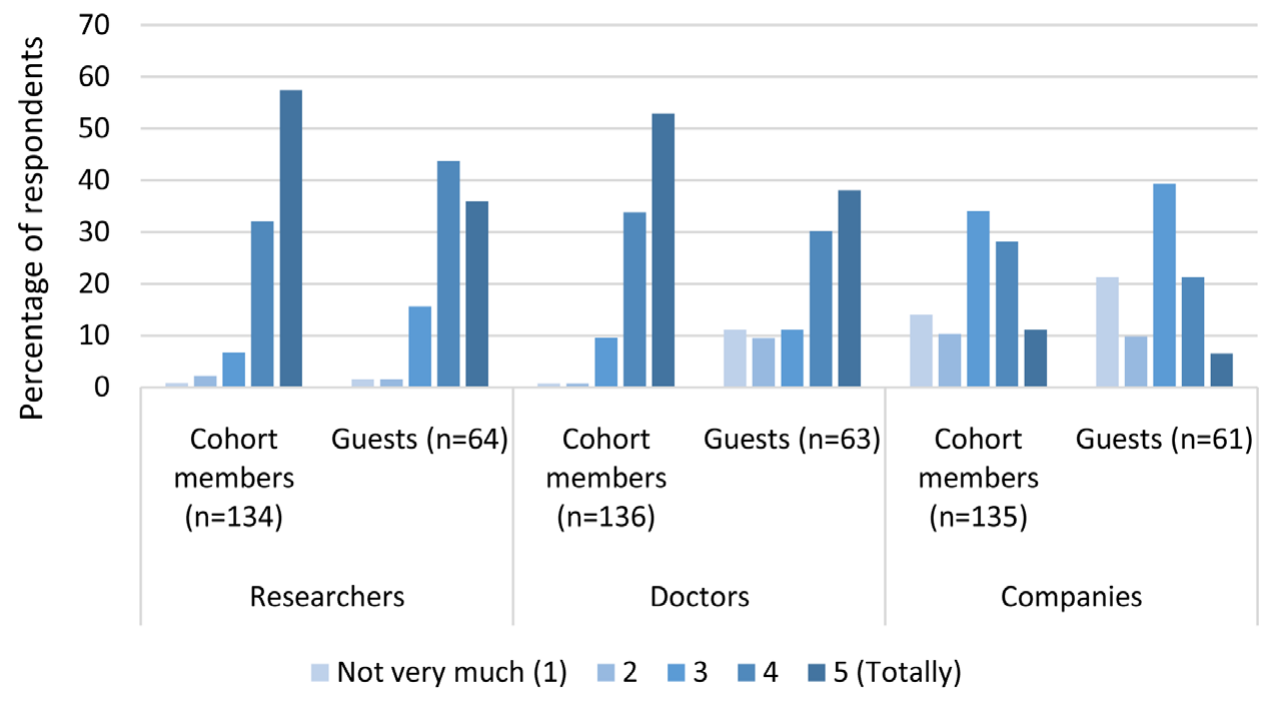
Trust in researchers, doctors and companies. Responses are divided into cohort members and guests. ‘I would rather not say’ responses are not shown (n ≤4). Only those who answered both Q1 regarding ‘cohort’ membership AND this question are included.

***Trust.*** A significant difference was observed between groups for trust in researchers (Mann-Whitney U = 3349, p=0.008) and doctors (Mann-Whitney U = 3110, p=0.001), such that cohort participants showed higher trust in researchers and doctors than guest participants ( Figure 2). This is perhaps to be expected, as cohort members have self-selected to participate in health research studies. Responses to trust in companies was not significantly different between groups (p=0.095).

**Figure 3.**
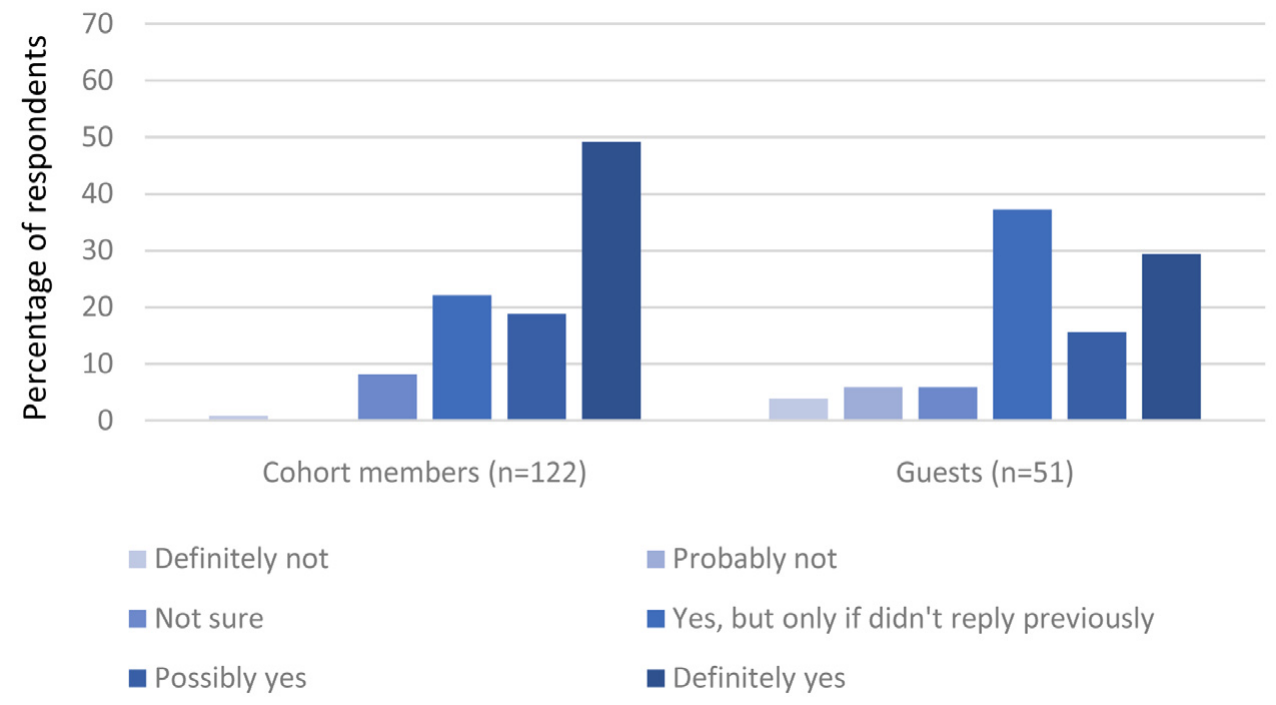
Should post-mortem brain donation be re-offered? Responses to “If someone has said no, or not given a reply [to post-mortem brain collection], should researchers approach them again to see if they have changed their mind/would like to donate now?” Divided by cohort members and guests. Only those who answered both Q1 regarding ‘cohort’ membership AND this question are included.

***Post-mortem brain donation.*** Guests were significantly less positive than cohort members about re-approaching someone to ask for their consent to donate post-mortem brain tissue (Mann-Whitney U = 2291, p=0.005; Figure 3).

**Figure 4.**
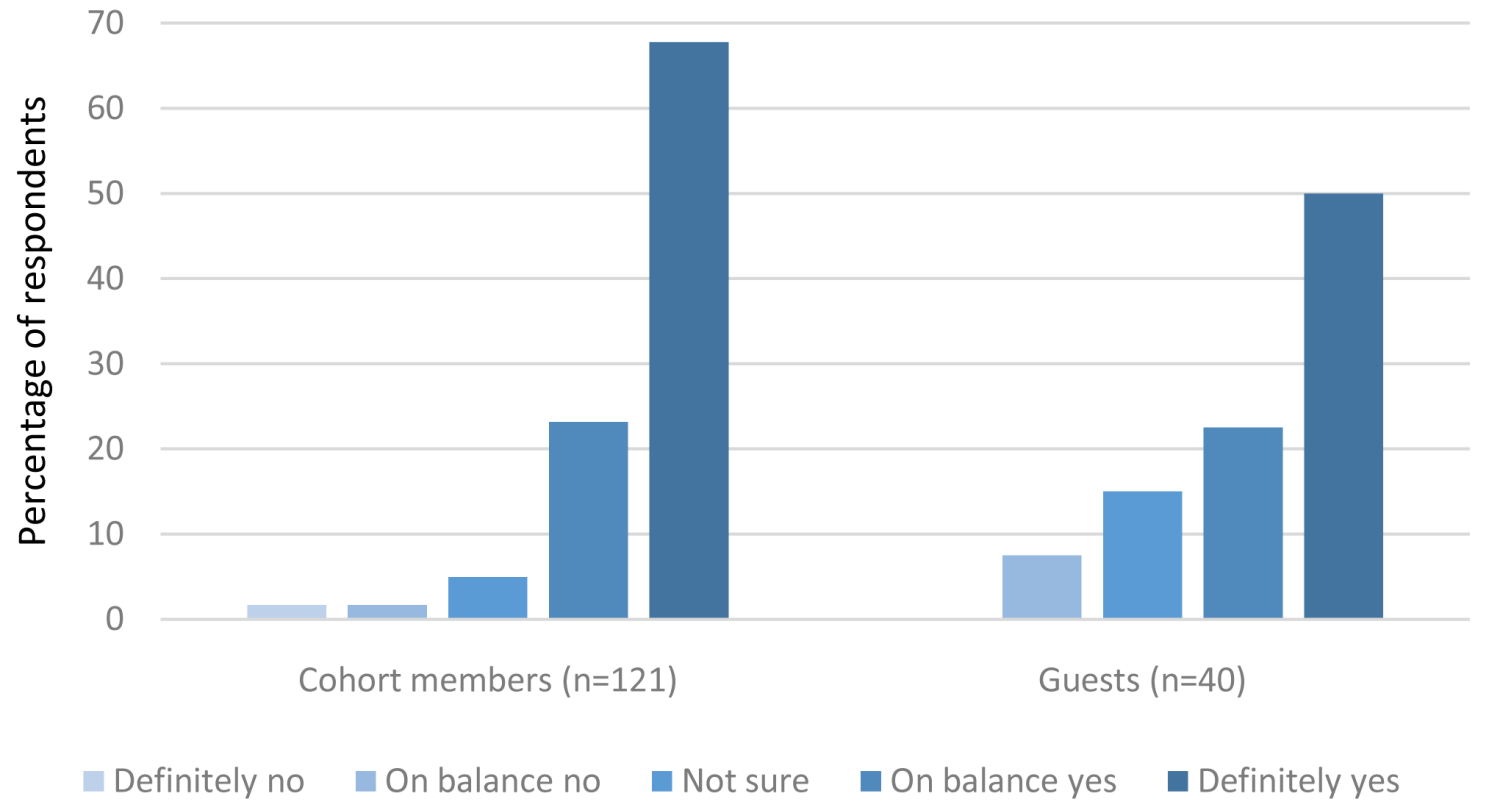
Willingness to undergo testing on a more frequent basis. Should testing be carried out on a more frequent basis (e.g. every two years)? Divided by cohort members and guests. ‘I would rather not say’ responses are not shown (n = 3). Only those who answered both Q1 regarding ‘cohort’ membership AND this question are included.

***Frequency of research testing.*** Guests were also significantly less positive about the possibility of more frequent research visits, compared with cohort members (Mann Whitney U = 1886, p=0.015; Figure 4). However, the phrasing of this question was not ideal, as guests had not undergone any previous testing. *[Question text: Would you be willing to repeat the testing you have already done but on a more frequent basis? (i.e. every 2 years?)]* In hindsight, a ‘not applicable’ option would have been useful for guests and may have captured more accurately the guest’s experience than the included ‘I’d rather not say’(2 reponses) and ‘not sure’ (6 responses).

## Discussion

The current results demonstrate the feasibility of interactively measuring public attitudes to research, including data linkage, through the use of live voting pads. The substantial majority of our audience were very positive about health data linkage and sharing. Most were prepared to consider new and more frequent forms of data collection such as the use of ‘Fit-bits’ and brain scans and were keen to encourage others to take part in research. Responses were less uniformly positive when the question of trust in commercial companies was posed, or when they were asked to consider taking a new drug or changing an aspect of their lifestyle.

Although our results are from a relatively small pool of people, larger scale public surveys corroborate several of our results. For example, The Wellcome Trust found that 77% of the UK public would be willing to share their anonymised medical records for the purposes of medical research ( Ipsos MORI, 2016).

### Trust

Unsurprisingly, given the high rates of audience participation in research, our participants showed a high level of trust in researchers and doctors. This result was echoed again in a recent Generation Scotland email survey ( Edwards *et al.*, 2019). Nonetheless, our findings are in line with previous research which reported that 92% of the UK public trust doctors to tell the truth and 85% trust scientists ( Ipsos MORI, 2018). Our more mixed response to trust in ‘commercial companies’ replicated previous reports of a lower level of trust in ‘business leaders’ (34% of those surveyed trusted them to tell the truth, Ipsos MORI, 2018).

In a similar 2016 survey, people trusted doctors and nurses to provide accurate and reliable information about medical research (64% of those surveyed trusted them ‘completely’ or ‘a great deal’). University researchers came a close second (59%). By contrast, a much lower degree of trust was expressed for ‘Pharma Scientists’ (32%) and ‘Industry Scientists’ (29%) ( Ipsos MORI, 2016), who were perceived to ‘exaggerate information’ and ‘only show positives’.

Using NHS data for ‘big-data’ research leads to significant ethical questions around access, privacy, confidentiality, trust and rights ( Adibuzzaman *et al.,* 2018). As researchers are increasingly encouraged to develop collaborations with industry, it is important to consider what steps could be taken to maintain trust and transparency, especially when working with public or donated data. These might include public consultations, or campaigns and collaborative knowledge exchange efforts which include a wide range of stakeholders.

### Policy implications – Guthrie Spot

Our data have implications for policy, by demonstrating strong support for research access to ‘Guthrie Cards’ (neonatal blood spots). This is in line with the results of a recent email survey by Generation Scotland ( Edwards *et al.*, 2019) and a more in-depth Citizens’ Jury, which was unanimous in its conclusion that research access to Guthrie cards was in the public interest, subject to appropriate ethical considerations, governance and oversight (June 2017, Porteous *et al*, in preparation, see also Edwards *et al.*, 2019). Nevertheless, there continues to be an embargo on the use of Guthrie spots for research in Scotland (and the rest of the UK), pending the conclusion of an ongoing stakeholder and public consultation.

### Age of consent

A key ethical debate for our birth cohorts concerns age of competence. What is the appropriate age at which to seek informed consent from children who were enrolled in a cohort study by their parents at birth? Our respondents were fairly equally split between 12, 14 and 16 years old.

Scottish law has specific rules which govern a child’s participation in clinical trials (i.e. the testing of a medicinal product). In these circumstances, consent must be given by a parent or legal representative for all children under 16 years of age ( NHS Health Research Authority, n.d). However, there is no such legal provision for other types of research. Instead, guidelines are offered by The Health Research Authority, which equate research consent with treatment consent ( Griffith, 2016); i.e. children under 16 have a right to consent to treatment (and by extrapolation, research) if they are deemed, by a medical practitioner, to be competent to do so ( HRA, n.d.). This competence depends heavily on the child’s capacity to understand the research being proposed and any risks that it entails. Furthermore, attempting to achieve consent from a child puts the onus on the researcher to present the information in an age-appropriate way that fosters true voluntary decision making ( HRA, n.d.). Conversely, if a child objects to participation, this is assumed to be their legal right, with case law suggesting that parents will not be able to overrule this.

Yet, even when an age is decided, this is just the beginning of the debate; what should happen to the data collected so far if a teenager withdraws their consent? Should they be offered an opportunity to ‘rejoin’ the study aged 16, 18 or 21? These and other questions will continue to be discussed and debated in the coming years.

### Public engagement with research

Finally, in line with all major UK funding councils, we believe that public engagement should be a priority for all research studies, especially cohort studies. For medical research, altruism is a key motivator, ( Jones *et al.,* 2016; National Institute for Health Research, 2019), but our participants also expect to benefit personally ( McCann *et al.,* 2010). This event brought together researchers and cohort members with a spirit of openness and community; to learn new things and engage with one-another. However, other methods such as circulating newsletters and sending birthday cards have also been used by these cohort studies to share results and build social bonds. Madsen *et al.* (1999) found that amongst former clinical trial participants who now held a negative attitude towards future participation, a common reason was the lack of information about results. Thus, feeding back research results in a clear and useful way was important not only for our own cohorts, but to the future of research participation in general.

### Strengths and limitations

A significant limitation is that the majority of our audience already participate in research and had chosen to attend a university-run knowledge exchange event. In addition, the majority of our participants came from the Lothian Birth Cohort, so are ~82 years old. Therefore, our results cannot be assumed to be representative of public opinion. Another methodological limitation is that not all participants answered every question. This is particularly pertinent for those who did not answer the first question (e.g. arrived late) and therefore could not be allocated to a cohort or labelled as a guest for further analysis. That said, the voting pad data collection method successfully served the dual purpose of improving engagement during the event and providing useful data for researchers to use in the future (e.g. on ethics forms and grant applications).

## Conclusions

Public engagement events that allow participants to express their opinions have value to both researchers and the general public. Using a simple voting pad system, we were able to collect data which will likely influence and facilitate our future research and public engagement efforts. We would encourage other researchers to consider how they might facilitate such two-way interactions during their own public engagement events. Our findings reveal that both research participants and their guests are broadly supportive of research access to the data and samples, albeit they are less supportive when commercial interests are involved.

## Data availability

### Underlying data

Edinburgh DataShare: A Celebration of Scottish Health Cohort Studies: Participant’s attitudes towards data research. https://doi.org/10.7488/ds/2728 ( Beange *et al*., 2019).

This project contains the following underlying data:

- Voting Pad Raw Results [xlsx]. Raw results from each participant.
- Voting Pad Results Summary (by group) v2 [xlsx]. Summary results given by group from all participants.

### Extended data

Edinburgh DataShare: A Celebration of Scottish Health Cohort Studies: Participant’s attitudes towards data research. https://doi.org/10.7488/ds/2728 ( Beange *et al*., 2019).

This project contains the following extended data:

- Cohort Event Invitation [pdf]. The event invitation sent to all cohort participants.
- Cohort Meeting Slide deck [pptx]. This file contains all slides shown during the meeting, including each of the questions asked of the participants.
- Video #1 Andrew Morris Introduction [mp4]. Video of the talk given by Andrew Morris.
- Video #2 Andrew McIntosh [mp4]. Video of the talk given by Andrew McIntosh.
- Video #3 James Boardman [mp4]. Video of the talk given by James Boardman.
- Video #4 Corri Black [mp4]. Video of the talk given by Corri black
- Video #5 David Porteous [mp4]. Video of the talk given by David Porteous.
- Video #6 Stephen Lawrie [mp4]. Video of the talk given by Stephen Lawrie.
- Video #7 Ian Deary [mp4]. Video of the talk given by Ian Dearie.
- Video #8 David Batty [mp4]. Video of the talk given by David Batty.
- CCACE Notes - Celebrating Participatory Research [pdf]. A ‘magazine’ that was given to each event attendee in their welcome pack. Each speaker has an article, and each cohort is described.
- Information Sheet – clicker [pdf]. Information sheet given to each participant.
- Event Feedback Form

Data are available under the terms of the Creative Commons Attribution 4.0 International license (CC-BY 4.0).
