# Supplementary material for: Using a knowledge exchange event to assess study participants’ attitudes to research in a rapidly evolving research context
Source: Wellcome Open Res. 2020 Aug 28;5:24. Originally published 2020 Feb 10. [Version 2] doi: 10.12688/wellcomeopenres.15651.2 (PMC7361507; doi:10.12688/wellcomeopenres.15651.2)
Supplement: Peer review history

# Peer review: Reviewer response for version 2

**DOI:** 10.21956/wellcomeopenres.17855.r40203

**Published:** 2020-09-01

**Authors:** Mhairi Stewart

**Affiliations:** 1 University of St Andrews, St Andrews, UK

No further comments to make.

Is the work clearly and accurately presented and does it cite the current literature?

Partly

If applicable, is the statistical analysis and its interpretation appropriate?

Yes

Are all the source data underlying the results available to ensure full reproducibility?

Yes

Is the study design appropriate and is the work technically sound?

Yes

Are the conclusions drawn adequately supported by the results?

Yes

Are sufficient details of methods and analysis provided to allow replication by others?

Yes

Reviewer Expertise:

Knowledge Exchange. Public Engagement. Institutional Culture Change. Science Communication.

I confirm that I have read this submission and believe that I have an appropriate level of expertise to confirm that it is of an acceptable scientific standard.

# Peer review: Reviewer response for version 1

**DOI:** 10.21956/wellcomeopenres.17148.r39112

**Published:** 2020-07-15

**Authors:** Justin Starren

**Affiliations:** 1 Feinberg School of Medicine, Northwestern University, Chicago, IL, USA

The study describes a real-time survey of cohort participants’, and their guests’, attitudes about research. The limitations of the work are driven mainly by selection bias and missing information. 

 All of the participants are closely connected with longstanding cohort studies. Unfortunately, the authors did not collect how long each participant was in the cohort, only which cohort. That said, because of the nature of the cohorts, some participants have been involved for over half a century. Keeping research subjects engaged is a huge problem. Any research subject who continues for over 50 years is not typical of the population. A valuable reference point would have been the overall percentage retention of the various cohorts. It is possible that these individuals are a highly selected fraction of the starting cohort, and that most participants already dropped out. Since each individual identified their cohort and guest could be separated from cohort participants, it seems surprising that among-cohort comparisons were not done. This would have strengthened the paper.

 The results were further biased by the fact that this was a gala celebrating the successes of cohort-based research. Therefore, the participants were presented with success stories, and then asked their opinions. The positive nature of the evening is likely to bias results to the positive. 

 The negative reaction of pharmaceutical research was striking. However, one is left to wonder whether results would be different had the gala been celebrating the many breakthroughs and lives saved by pharmaceutical companies.

 Overall, the work is sound in terms of the conclusions drawn from the data. However, the selected population is so narrow and atypical that few, if any, generalizations can be made from the results to the general population. We are left with the unsurprising conclusion that people who have chosen to participate in research for decades are positively disposed toward research. 

 Stylistically, the paper could combine figures and tables more efficiently. For examples, tables 3 and 4 could be combined so that questions and answers are adjacent. Similarly, figure 2 could have been incorporated into the table as “sparkline” bar charts to provide graphical and numerical data side by side. Figures 3 and 4 present comparisons between cohort members and guests differently. Figure 3 presents separate graphs for each group while 4 mixes the groups and uses color coding.

Is the work clearly and accurately presented and does it cite the current literature?

Yes

If applicable, is the statistical analysis and its interpretation appropriate?

Yes

Are all the source data underlying the results available to ensure full reproducibility?

Yes

Is the study design appropriate and is the work technically sound?

Partly

Are the conclusions drawn adequately supported by the results?

Yes

Are sufficient details of methods and analysis provided to allow replication by others?

Yes

Reviewer Expertise:

Biomedical Informatics and Translational Research. I have done multiple studies bringing technology into the community and assessing attitudes. I have used live-response technology.

I confirm that I have read this submission and believe that I have an appropriate level of expertise to confirm that it is of an acceptable scientific standard.

# Peer review: Reviewer response for version 1

**DOI:** 10.21956/wellcomeopenres.17148.r39261

**Published:** 2020-07-14

**Authors:** Becky Hothersall

**Affiliations:** 1 University of Glasgow, Glasgow, UK

This is a useful article which contributes to knowledge and understanding of the contribution public engagement can make to research and to society.

 The introductory literary review provides helpful context and justification of the issues explored. Citizens’ juries are mentioned later in the policy implications section and I would like to have seen a brief mention earlier of these here, perhaps to assess the comparative strengths of the two approaches.

 The methods and statistical approaches are sound and are adequate to address the questions asked. The data provided are useful and inclusion of detailed logistical considerations is helpful for understanding of process and for those wishing to replicate the methods. As such, I appreciated the level of detail provided about the questions asked; these were appropriate and clear. The event clearly generated high levels of audience engagement (indicated by numbers answering questions). Questions were sufficiently sensitive to detect differences between groups and questions, such as the apparent negative influence of commercial interests on audience trust. The use of Likert scales with neutral points and clear options follows good assessment practice.

 I felt the authors dealt well with the balance between collecting nuanced data, placing a burden on participants (it was important that the event was interesting and beneficial to them) and research ethics. For example, it would have been fascinating to explore paired data (cohort participants and their guests) or understand more about demographic influences, but this would have required a more intrusive approach. Nevertheless, the unpaired data from this event generated some interesting findings and confirm the utility of this general approach such that future studies could delve deeper as appropriate to their audience and research questions. There is also scope for these methods to be used to elicit more detailed information or ideas from participants. This could contribute to greater control or ownership of research by patients and members of the public, a stated goal for several funders.

 The authors acknowledge that participation in research probably contributed to the audience’s high trust and interest in health research. I agree with the first reviewer that this bias is likely to extend to the guest group. It is still valid to compare their responses and interesting that attitudes were consistently more positive in the cohort group. The authors are careful not to attribute causality to the differences found. However, I feel that the difference in attitude towards more frequent testing (q14) could be explained by guests (who have not participated in testing) being unsure how to answer. A number selected “not sure”, which they may have used in the absence of a “Not Applicable” option.

**Minor comments on question set, analysis and results**

 The authors are explicit that corrections for multiple comparisons were not included. It would have been good to flag this in the discussion when assessing the potential significance of the differences between the cohort and guest groups.

 They could also have acknowledged that the Lothian Birth cohort contributed by far the highest percentage of participants. Presumably this group has a much older age range than most cohorts and may therefore be less generalizable.

 For Figs 2, 4 and 5, it would be helpful to include an n= in corner of graph for ease of reference (this info can be obtained from tables).

 For the brain donation question, it would have been interesting to understand the context further. Were people being asked about a scenario several years later, or soon after the research?

**Other minor comments**

 To contribute to the purposes of learning from the outputs of the event, it would be helpful to include in the supplementary information a copy of the feedback form used.

 My assessment of accuracy is subject to the discrepancy already noted by the first reviewer.

 Typo in Abstract: Background, line 2 – replace “who” with “which”?

Is the work clearly and accurately presented and does it cite the current literature?

Yes

If applicable, is the statistical analysis and its interpretation appropriate?

Yes

Are all the source data underlying the results available to ensure full reproducibility?

Yes

Is the study design appropriate and is the work technically sound?

Yes

Are the conclusions drawn adequately supported by the results?

Yes

Are sufficient details of methods and analysis provided to allow replication by others?

Yes

Reviewer Expertise:

Public engagement, knowledge exchange, technology enhanced learning.

I confirm that I have read this submission and believe that I have an appropriate level of expertise to confirm that it is of an acceptable scientific standard.

# Peer review: Reviewer response for version 1

**DOI:** 10.21956/wellcomeopenres.17148.r38565

**Published:** 2020-05-13

**Authors:** Mhairi Stewart

**Affiliations:** 1 University of St Andrews, St Andrews, UK

This article describes the outputs, and their analysis, of a knowledge exchange event with five different cohorts of longitudinal cohort studies and guests including friends and relatives of cohort participants. The aims were to understand attitudes and values around the potential of using record linkage and material sources (e.g. blood spots or brain scans) in research activity.

 The authors describe the methodology thoroughly and very helpfully. The findings are thoroughly described, although this reviewer has found some discrepancies in figures which are listed below. 

 In terms of conclusions, the findings for trust and the use of medical records agree with the findings in other similar studies. The authors have identified a limitation in that the participants are primarily within research cohort studies and are therefore more likely to be positive in their attitudes. They do suggest that the non-cohort participants may be more indicative of a general population, however I feel that as friends and relatives of the cohort participants, this might in fact still be less representative of a general population audience.

 In all, this is a useful paper on the methodology with some insights the value of dynamic two-way communication channels in the delivery of events of this type.

 Suggestions:

 Table 5. I feel it is worth reiterating here why the total number in the tests do not add up to the total numbers of respondents in Table 4 as it was confusing and required some backtracking to understand.

 Errors: Page 12 contains what seem to be typographical errors in number reporting:

- Para 4 line 1. ‘For research data collection purposes 78% of our respondents were willing…’ From Table 4 this figure should be 88%. Note this error is reflected in the abstract.
- Para 4 line 16. ‘Nevertheless, 68% of our respondents…’ From Table 4 this figure should be 78%.

Is the work clearly and accurately presented and does it cite the current literature?

Partly

If applicable, is the statistical analysis and its interpretation appropriate?

Yes

Are all the source data underlying the results available to ensure full reproducibility?

Yes

Is the study design appropriate and is the work technically sound?

Yes

Are the conclusions drawn adequately supported by the results?

Yes

Are sufficient details of methods and analysis provided to allow replication by others?

Yes

Reviewer Expertise:

Knowledge Exchange. Public Engagement. Institutional Culture Change.

I confirm that I have read this submission and believe that I have an appropriate level of expertise to confirm that it is of an acceptable scientific standard.
